# An Unusual Clinical Outcome and Surgical Intervention for Mediastinal Histoplasmosis

**DOI:** 10.7759/cureus.30977

**Published:** 2022-11-01

**Authors:** Jessica Katsiroubas, Sebron Harrison

**Affiliations:** 1 General Surgery, NewYork-Presbyterian Brooklyn Methodist Hospital, Brooklyn, USA; 2 Thoracic Surgery, NewYork-Presbyterian Brooklyn Methodist Hospital, Brooklyn, USA; 3 Cardiothoracic Surgery, Weill Cornell Medicine, New York, USA

**Keywords:** posterior mediastinal mass, mediastinal masses, video-assisted thoracic surgery, thoracic surgery, histoplasmosis

## Abstract

*Histoplasma capsulatum*, an endemic mycosis in the United States, can result in a variety of clinical manifestations. When encountering histoplasmosis sequelae, such as mediastinal adenitis, granulomas, and fibrosing mediastinitis, surgical management may be an unexpected but necessary treatment. Due to the complexity of the mediastinum, it is imperative to have optimal operative planning. In this report, we present an unusual case of an inflammatory mediastinal mass in the setting of acute histoplasmosis resulting in left atrium compression and arrhythmias.

## Introduction

*Histoplasma capsulatum*, an endemic mycosis in the United States, can present with a variety of clinical manifestations. Inhalation of airborne particles in the conidia state most commonly results in asymptomatic colonization of the lungs, though exposure can also trigger a robust inflammatory response manifesting with flu-like symptoms such as fever, shortness of breath, cough, and weight loss [1-3]. In most cases, acute pulmonary histoplasmosis is self-limiting. However, when required, treatment varies depending on the severity of the illness and the immune status of the patient. An oral regimen of itraconazole (six to 12 weeks) is the therapeutic typically prescribed when symptoms persist [4]. In the rare instance when histoplasmosis-related sequelae or complications develop, surgical intervention may be warranted with the goal of alleviating compression on thoracic and mediastinal structures or relieving obstruction [5]. In this report, we present an unusual case of an inflammatory mediastinal mass in the setting of acute histoplasmosis resulting in left atrium compression and arrhythmias.

## Case presentation

**Figure 1:**
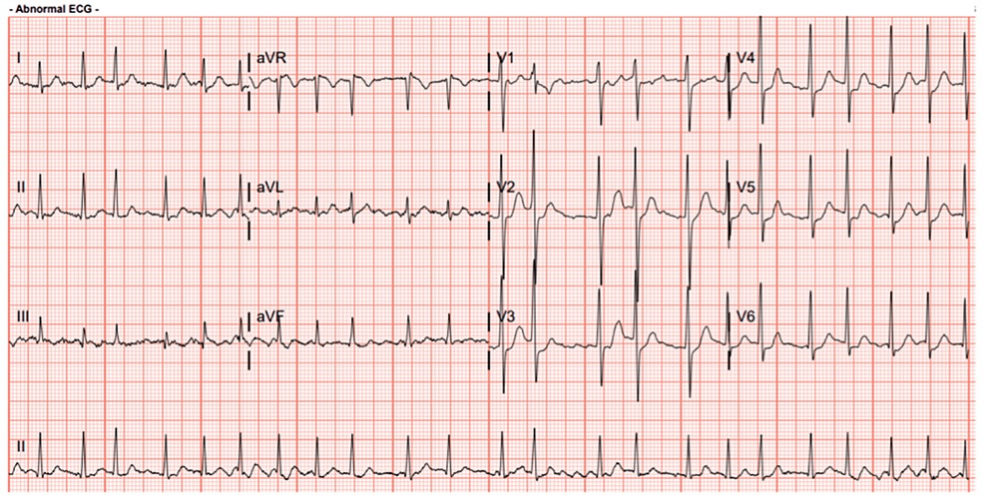
Electrocardiogram Electrocardiogram notable for atrial fibrillation with a rapid ventricular response. Rate: 136; PR: 0; QRSd: 90; QT: 278; corrected QT: 420.

A 33-year-old male with a history significant for a thyroid nodule presented with periods of palpitations and a chronic cough following a cave diving trip. The initial workup consisted of medical management for suspected bronchitis and new-onset atrial fibrillation (Figure 1). The patient experienced debilitating palpitations and lightheadedness requiring several visits to the emergency room. The patient underwent radiofrequency ablation by wide area circumferential ablation (WACA) to isolate the pulmonary veins and ablation of the cavotricuspid isthmus (CTI) to create a bidirectional CTI block, which briefly stopped the arrhythmias.

Nevertheless, the cough lingered, and the palpitations reoccurred with increasing severity. Pulmonary function testing was obtained indicating a fixed upper airway obstruction. Imaging was obtained, which identified a 3.4-cm subcarinal soft tissue density on computed tomography (CT). The patient underwent bronchoscopy with a biopsy of a subcarinal lymph node, which revealed necrotic debris and the presence of scant organisms identified with gram stain consistent with *H. capsulatum*. Treatment was begun with itraconazole; however, debilitating episodes of palpitations and lightheadedness requiring recurrent visits to the emergency room persisted.

Cardiac magnetic resonance imaging (MRI) was obtained to further evaluate the mass. The MRI depicted a large posterior mediastinal mass located posterior to the right upper pulmonary vein, abutting and compressing the superior-posterior aspect of the left atrium. Cardiac electrophysiologists conferred the location of the mass was likely a nidus for the arrhythmias. With evidence of cardiac compression and persistence of symptoms despite medical therapy, the patient was taken to the operating room for exploration, excision of the mass, and to ensure the absence of additional pathology.

The procedure began utilizing a right video-assisted thoracoscopic approach (VATS) and a large inflammatory mass was identified in the subcarinal space. A meticulous dissection of the mass was started in an attempt to visualize the extent of the space-occupying lesion. However, due to the inability to adequately visualize the posterior mediastinal space, the procedure was converted to a thoracotomy via a left posterolateral incision. With improved visualization, the mass was found adherent to the right inferior pulmonary vein, esophagus, pericardium, and right lung. Following continued meticulous dissection, the mass was successfully removed, and no additional pericardial compression was observed.

Postoperatively, the patient recovered uneventfully in the cardiothoracic intensive care unit and was discharged with itraconazole on postoperative day three. Final pathology demonstrated necrotizing granulomatous inflammation, a sequela of pulmonary histoplasmosis. Subsequently, the patient returned home and remains symptom-free at one-year follow-up with no resultant complications or readmissions.

## Discussion

*H. capsulatum* is an endemic mycosis in the Mississippi and Ohio river valleys with an estimated incidence of 6.1 cases per 100,000 individuals [1]. Although most commonly manifesting with flu-like symptoms, mediastinal manifestations including adenitis, granulomas, or fibrosing mediastinitis are all potential complications that can lead to obstruction and compression of mediastinal structures requiring the need for surgical intervention. Most commonly, symptomatic presentations are the result of airway compression [1]. Hammoud* *et al. studied complications from pulmonary histoplasmosis and found that hemoptysis and recurrent pneumonia were the main reasons for operative intervention [5]. In our patient, the development of left atrial compression and atrial fibrillation represents a unique sequela of histoplasmosis.

The complexity of the mediastinum makes imaging a necessity for diagnosis as well as accurate delineation of mediastinal structures and compartments. When concerned for mediastinal pathology, the cross-sectional images generated by CT are useful in detecting cysts, adipose tissue, soft tissue masses, calcification, and air, which can aid in the creation of a differential diagnosis [6]. MRI, although used less frequently, offers the ability to more clearly visualize the structures of the mediastinum, and should especially be used when concerned for vascular involvement [6]. In our case, CT imaging demonstrated a 3.4-cm subcarinal soft tissue density; however, in the setting of a persistent cardiac arrhythmia, MRI was obtained to further characterize the mass and assist in operative planning.

Utilizing a VATS approach to mediastinal surgery offers the benefits of decreased morbidity and recovery time in comparison to traditional thoracotomy [7]. However, exploring the confined space of the posterior mediastinum can also limit the utility of VATS [8]. A narrow working field with adherent or friable inflammatory tissue can present grave difficulty for the surgeon. In this case, the dense inflammatory tissue of the subcarinal mass limited the visual field requiring the creation of a left posterolateral thoracotomy. Broadened exposure enabled visualization of the mass’s adherence to the right inferior pulmonary vein, esophagus, pericardium, and right lung, thus facilitating a successful surgery.

## Conclusions

In conclusion, histoplasmosis can result in a variety of clinical manifestations. When encountering histoplasmosis sequelae, such as mediastinal adenitis, granulomas, and fibrosing mediastinitis, surgical treatment may be an unexpected but necessary treatment. Due to the complexity of the mediastinum, it is imperative to have optimal operative planning. When dense inflammatory tissue is encountered, especially in tight areas such as the posterior mediastinum, early conversion to an open thoracotomy should be considered to improve surgical field exposure and facilitate a safe completion of the operative procedure.
